# The objective assessment of cough frequency: accuracy of the LR102 device

**DOI:** 10.1186/1745-9974-7-11

**Published:** 2011-12-01

**Authors:** Sophie Leconte, Giuseppe Liistro, Patrick Lebecque, Jean-Marie Degryse

**Affiliations:** 1 Institute of Health and Society, Université Catholique de Louvain (UCL),Clos-Chapelle-aux-Champs 30 bte 3015 Brussels, Belgium; 2 Department of Pulmonology, University Hospital St.Luc, Université Catholique de Louvain (UCL), Avenue Hippocrate 10, 1200 Brussels, Belgium; 3 Department of Pediatrics, Cliniques Universitaires Saint-Luc, Université Catholique de Louvain (UCL), Avenue Hippocrate 10, 1200 Brussels, Belgium; 4 Department of General Practice, Katholieke Universiteit Leuven, Kapucijnenvoer 33, bus 7001, 3000 Leuven, Belgium

**Keywords:** cough, validation study, cough meter

## Abstract

**Background:**

The measurement of cough frequency is problematic and most often based on subjective assessment. The aim of the study was to assess the accuracy of the automatic identification of cough episodes by LR102, a cough frequency meter based on electromyography and audio sensors.

**Methods:**

Ten adult patients complaining of cough were recruited in primary care and hospital settings. Participants were asked to wear LR102 for 4 consecutive hours during which they were also filmed.

**Results:**

Measures of cough frequency by LR102 and manual counting were closely correlated (r = 0.87 for number of cough episodes per hour; r = 0.89 for number of single coughs per hour) but LR102 overestimated cough frequency. Bland-Altman plots indicate that differences between the two measurements were not influenced by cough frequency.

**Conclusions:**

LR102 offers a useful estimate of cough frequency in adults in their own environment, while significantly reducing the time required for analysis.

## Introduction

Cough is a common reason for seeking medical care [1,2]. Not only is it a prevalent problem, it also has a significant impact on quality of life [3-6]. Yet it has not been extensively studied using objective measures. The evaluation of a cough, in clinical practice but also in most clinical trials, is usually based on patients' subjective assessment.

The studies that have used objective measurements have reported inconsistent correlations between objective and subjective measurements [7-19]. The following assessment tools are currently available: cough monitors used to objectively measure cough frequency, and quality-of-life questionnaires, verbal descriptive scores (VDS), and visual analogue scales (VAS) used to subjectively measure cough severity. The assessment of cough severity can involve several different aspects: (1) the importance of the cough, objectively measured in terms of the frequency or intensity of the expulsive effort (2) the impact of the cough on the patient's quality of life and (3) the importance of the cough as perceived by the patients [20]. In a recent systematic literature we reported the psychometric characteristics of three validated cough-specific quality-of-life scales (Leicester Cough Questionnaire, Cough Quality of Life Questionnaire, and Burden of Cough Questionnaire). However we found no validation studies on VDS or VAS. The correlations between quality-of-life scores and cough frequency were good suggesting that cough frequency has an impact on the patient's quality of life even though other elements are most likely to influence the quality-of- life scores. The correlations between VDS or VAS and more objective methods, such as cough frequency monitoring varied considerably. Patient's perceptions of the importance of their cough when measured by VDS or VAS may be influenced by elements other than cough frequency. So despite having good face validity, these scores and scales cannot be regarded as validated tools indicative of cough frequency or the repercussion of the cough on the patient's quality of life. We concluded that cough-specific quality-of-life questionnaires can provide valid outcomes for research into cough and that although the current developments in cough monitoring devices are promising, further studies on a large scale under more realistic conditions are required before they can be recommended for widespread use [20]. However an important requisite for this is a device that can monitor in an accurate way cough frequency in humans.

Objective accurate cough measurement could be used as an important tool in some clinical situation such as monitoring a therapeutic trial in patients suffering of chronic cough as well for research purpose to judge the efficacy of cough suppressant. The use of cough suppressants is substantial. Every week in the United States, 4% of children take cough suppressants [21], despite the fact that evidence as to their effectiveness on valid outcomes is lacking, and that potentially serious side-effects have been reported [22]. Most studies of currently available cough suppressants have used non-validated subjective measurements of cough severity such as visual scales and descriptive scores [22,23].

Measuring cough objectively would not only improve the quality of clinical trials for cough suppressants but could also further our understanding of the diagnostic value of cough characteristics such as frequency, intensity and timing.

LR102 is a cough frequency meter designed by Logan Sinclair [24,25]. It is based on the combined analysis of electromyography (EMG) signals from intercostal muscles and auditory signals. The accompanying software that was developed by the manufacturer to perform an off-line analyse of the registered signals, provides automated identification of cough *episodes*. This device has three potential advantages: 1) it can distinguish between the patient's coughs and those from others nearby, 2) it provides automated analysis, and 3) the equipment is compact, leaving patients free to pursue their normal activities in their own environment. Although it has already been used in various studies [15,18,19,24-27], it has only been validated for adults patients in one study in 1994 [24]. The validation study included only 4 adult patients and was limited to manual trace reading of cough episodes. The automatic identification of cough episodes has not been validated.

The purpose of this study was to assess the validity of the automated measurement of the frequency of cough episodes by the LR102 compared to manual counting of episodes viewed on video recordings.

This study was conducted to pilot the use of the cough meter in a larger diagnostic study, which was approved by the local ethics committee. The diagnostic study aimed to evaluate the predictive values of clinical signs and symptoms in cases of prolonged cough.

## Methods

### Patients

Patients complaining of coughing were recruited from inpatient (pulmonology) and outpatient settings, irrespective of their diagnosis. Participants had to be over the age of 16 and, for inpatients, in a single room. Informed consent was obtained from all participants.

### Cough recording

The patients' coughs were recorded simultaneously by two measurement methods: video recording and LR102. Recordings lasted for four hours. Most of the measurements were conducted during daytime; one was performed during the night.

#### LR102

LR102 is a multi-parametric device connected to the thoracic wall by 3 EMG sensors and a sound sensor. The EMG electrodes are placed as follows: one in the sixth right intercostal space, one in the left mid-clavicular area, and one at the epigastrium. The sound sensor is placed in the second left intercostal space.

**Figure 1:**
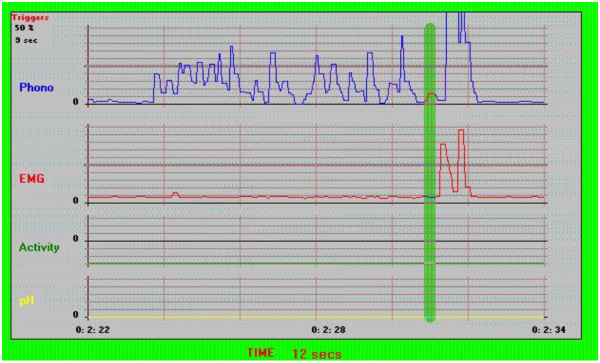
**Screen shot of the off line analysis program that allows automated measurement of the *cough episodes***. The LR102 is based on the combined analysis of EMG signals from intercostal muscles and auditory signals. A cough episode is identified when two events occur simultaneously: 1) there is a contraction (EMG burst) of the intercostals muscles, and 2) There is a sound of predetermined intensity. Thresholds for the intensities of the EMG and audio signals can be configured.

A cough is identified when two events occur simultaneously: 1) there is a contraction of the intercostal muscles, and 2) there is a sound of predetermined intensity. Data from the sensors can be displayed as a graph on the user interface: one band corresponds to variations in intensity of the sound signal, a second to variations in the intensity of the contractions of the intercostal muscles, and a third to base muscle activity as measured at the epigastrium. The following settings were used for the automated identification of cough episodes: intensity threshold of muscle contraction and audio signal at 70% of the maximum intensity, free interval of 2 seconds between two episodes (Figure 1).

#### Reference recording

The gold standard used in studies of cough meter accuracy is usually video recording. Audio recordings have also been used but are less reliable in distinguishing coughs from other persons nearby. A laptop with a webcam was placed in the room (hospital room or in the participant's home) with the patient. The patient was asked not to leave this room, but otherwise to pursue his/her normal activities.

#### Outcomes

Objective measurements of cough have used various units of measurement, i.e. number of cough episodes, number of single coughs, number of seconds during which there is coughing. Cough episodes may consist of a single cough or of a fit of coughs, and may be brief or long. The clinical significance of these differences is likely important. Indeed measurements of the number of seconds spent coughing or of the number of single coughs have been found to be more closely correlated with a quality of life score than that of the number of cough episodes [28]. Furthermore, it is possible that cough suppressants may reduce the length of cough episodes and the number of coughs within each episode without affecting the total number of episodes. We therefore chose to study both the number of single coughs and the number of coughing episodes. However LR102's automated output only indicates the number of coughing episodes. Number of single coughs was ascertained through viewing LR102 traces.

The following criteria were used:

1) Cough episode

### Video recording

A cough episode was defined as one or more explosive sounds, typical of a cough, following a deep inspiration with an interval of less than 2 seconds between successive components. Throat clearing and sneezes were excluded.

### Reading of LR102 traces

A cough episode was defined as a single cough or a series of coughs in which there was an interval of less than 2 seconds between successive components

2) Single coughs

### Video recording

Each expulsion of air accompanied by a sudden sound was defined as a "single cough".

### Reading of LR102 traces

The cough episodes identified were analysed to determine the number of single coughs within each episode. Audio peaks with an intensity in excess of 70% accompanied by a muscle contraction were considered single coughs, as were smaller peaks if they occurred within less than two seconds of other peaks and were accompanied by a muscle contraction. It should be noted that several peaks occasionally coincided with a single large muscle contraction. These were considered as distinct coughs within the same episode.

#### Analysis

The traces were read consecutively during two afternoons without consulting the videos. A flaw in the clock was detected, and was corrected on an hourly basis. The internal clock of the device (and therefore of the registration on the memory card) appeared to run 2 minutes/hour ahead as compared to a validated chronometer and as compared to the video recording.

Each video recording was watched in full by the same researcher (SL) in real time (i.e. without fast-forwarding the recording). The researcher was blinded to the results of LR102's automated analysis. The results are presented in terms of number of cough episodes per hour and number of single coughs per hour.

Regression and correlation analyses were conducted using SPSS^® ^version 16 for validation of the cough count assessed by cough meter. The mean difference between the two measurement methods was also computed. A Bland-Altman plot was generated using Medcalc^® ^and completed by an intraclass coefficient correlation for assessing the reliability.

## Results

A total of 40 hours of recording were analysed. Half the patients were inpatients. Most were young adults and the gender distribution was equal (Table 1).

**Table 1:** Participant characteristics

**Diagnosis**	n	95%CI
Cystic fibrosis	4	

Viral infection	5	

Other	1	

**Setting**		

Inpatients	5	

Outpatients	5	

**Age **		

Median (IQR)	33.5	30-57

**Cough frequency**		

Average number of cough episodes per hour according to LR102	22.57	15.47-29.67

Average number of cough episodes per hour counted on video	18.77	11.84- 27.70

Average number of single coughs per hour counted on LR102 traces	65.22	45.53- 84.92

Average number of single coughs per hour counted on video	52.67	32.54- 72.80

The cough meter was well tolerated by all but one patient who complained of itching at the electrode sites.

**Figure 2:**
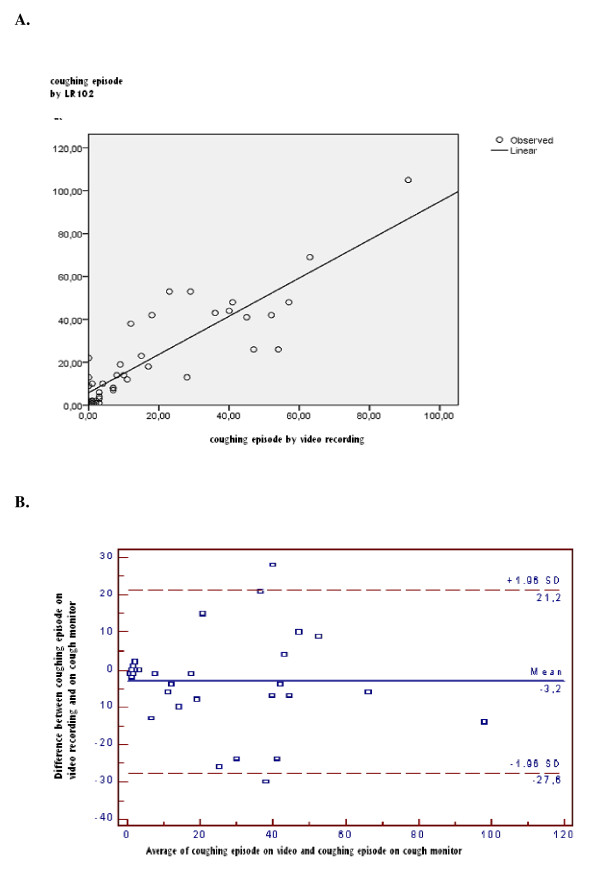
**The measurements of *coughing episodes *provided by the LR102 and manual counting based on video recordings (The unit of analysis is the number of episodes/hour)**. A. Scatter plot of the relationship between the two measurements. B. The Blant-Altman plot shows the difference of the number of *cough episodes *per hour between the two kinds of measurement in relation to the frequency of the cough episodes.

**Figure 3:**
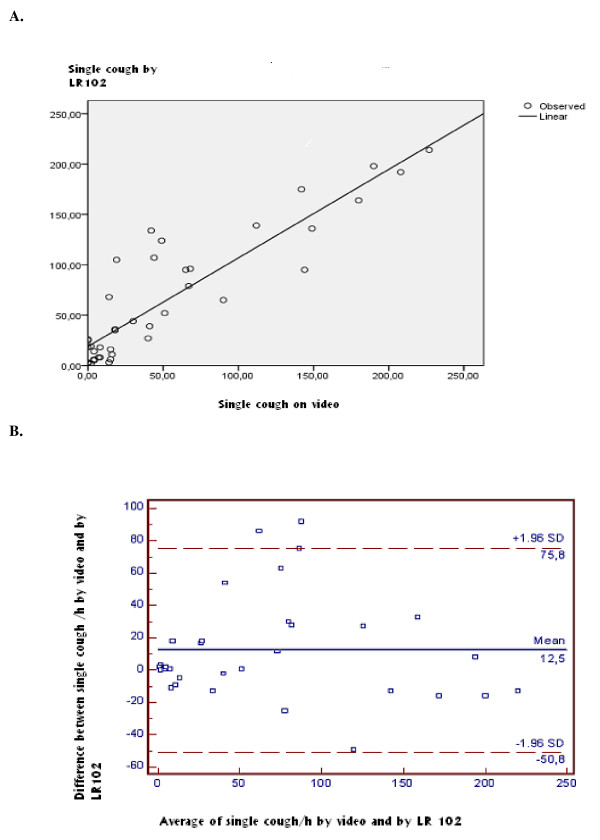
**The measurements of *single coughs *provided by the LR102 and the manual counting based on the video recordings (The unit of analysis is the number of single coughs/hour)**. A. Scatter plot of the measurements. B. The Bland-Altman plot shows the difference of the number of *single coughs *per hour between the two kinds of measurement in relation to the cough frequency.

The scatter plots (Figures 2a and 3a) indicate a linear association between the two measurements of cough frequency. The two methods produced cough frequencies that were closely correlated (r = 0.87 for number of cough episodes per hour; r = 0.89 for number of single coughs per hour). However the number of coughs per hour measured by the cough meter was higher than that measured by counting coughs on the video recording (number of cough episodes per hour: 22.57 vs 18.77; number of single coughs per hour: 65.22 vs 52.67). The difference was statistically significant. The mean difference between the two methods was 3.8 for cough episodes per hour (p = 0.04) and 12.5 for single coughs per hour (p < 0.01).

The number of single cough constituting one coughing episode varied from 1 to 40. The average was 3.4 (SD: 2.3) single cough per episode.

Bland-Altman plots indicate that there is no systematic difference between the two measures across the spectrum of cough frequency (Figures 2b and 3b). Intra class correlation coefficients were also good (ICC = 0.86 for episode (CI 95%= 0.75-0.92) and 0, 88 for single cough (CI 95%= 0.78-0.93)).

## Discussion

### Main findings

LR102 produces an accurate if slightly inflated measures of cough frequency. Reliability is consistent over the spectrum of cough frequency observed in this study. Furthermore, the device appears to be well tolerated. The overestimation of cough frequency may be due to occasional environmental noise occurring while the subject moves.

### Strengths and limitations

This validation study compared LR102 to the currently accepted gold standard. Measures were taken in both ambulatory and hospital settings, during the day and night time, and in patients with different diagnoses. Although participants were restricted to a room, they were able to move around freely and to develop usual activities. However large scale outdoor testing in the future seems mandatory. The measurement of cough episode frequency by LR102 was automated and could not as such be influenced by knowledge of results from the videos. The measurement of single cough frequency on the other hand was determined by trace reading by the same researcher who viewed the video recordings. It is however unlikely that she could recall recordings in any detail when analysing LR102 traces, especially since all traces were viewed consecutively. We believe, therefore, that this factor had little influence on results.

Our study does not enable analysis according to disease. The aetiology of a cough may influence characteristics such as timbre, intensity, pattern of coughing episodes (i.e. fits versus isolated coughs). The effect of such characteristics on the reliability of the device is unknown. A wide variation in types of cough from one patient to another was observed on the video recordings, but the small size of the sample precludes us from determining whether aetiology played a part in these variations.

Finally, the cross-sectional nature of the study means that longitudinal intra-patient analysis was not possible. This may be an avenue for further research to test the sensitivity of the device to change.

### Implications for practice and research

Were more widespread use of the device envisaged, further improvements should be considered, such as automated identification of single coughs and optimal timer calibration. False-positive rates could be reduced by improving the computerised analysis of cough pattern, thus allowing a distinction to be made between actual cough episodes and other noise, especially for ambulatory patients. Furthermore, the cost of the device should be reviewed.

Other devices exist. They fall into three broad categories. The first includes devices based solely on audio data. Traditional sound recordings [29] were initially improved by software aimed at directing researchers to relevant parts of the recordings, thus limiting time for analysis. Recently, specialised software have been developed allowing automatic identification of sounds consistent with coughing. The Leicester cough monitor [30] and vitaloJAK are two such devices. A second type of device detects coughs based on analysis of transmitted thoracic vibrations [31]. The third approach integrates audio data and data from motion sensors (LifeShirt system) [32]. The LifeShirt has been validated in comparison to a gold standard in 8 adult patients with chronic obstructive pulmonary disease. Further validation studies in outpatient settings, for other diseases, and in children should be conducted. Issues such as the shirt's weight and cost should also be addressed.

At this stage, it is not possible to determine whether motion analysis or EMG sensors obtain the best results. Specialised audio cough recognition software might also be a useful adjunct to either one of these types of devices. However we wonder if the idea of recording and analysing acoustic signals to monitor a single cough or cough episode should not be abandoned. The use of multiple acceleration and motion sensors might lead to more reliable monitoring devices.

## Summary

The difficulty in these studies was the limitation of activities because of the video recording. Further studies could use only portable audio recording as reference standard and permit 24 h recording.

The LR102 appears to be a feasable method of measurement of cough frequency in adults and could be a useful tool in practice as well as outcome in trial. However further studies according to disease and over the course of an illness are needed before recommending it as a reference tool in clinical trials.

## Competing interests

The authors declare that they have no competing interests.

## Authors' contributions

SL and JD drafted the manuscript. JD, SL and GL initiated study, and are responsible for its design. SL carried out all of the patient observations. All authors participated in the critical revision of the manuscript and red and approved the final manuscript.
